# Adaptation Mechanisms of Understory Vegetation in Subtropical Plantations: Synergistic Drivers of Stand Spatial Structure and Soil Fertility

**DOI:** 10.3390/plants14223452

**Published:** 2025-11-11

**Authors:** Fenglin Zheng, Dehao Lu, Wenyi Ou, Sha Tan, Xiongjian Xu, Shucai Zeng, Lihua Xian

**Affiliations:** 1 College of Forestry & Landscape Architecture, South China Agricultural University, Guangzhou 510640, China; 2 College of Horticulture and Landscape Architecture, Zhongkai University of Agriculture and Engineering, Guangzhou 519080, China; 3 Foshan Yunyong Forest Farm, Foshan 528518, China

**Keywords:** understory vegetation, biodiversity, biomass, structural equation model, south China’s plantations

## Abstract

Understory vegetation plays a pivotal role in enhancing forest biodiversity, and its restoration is crucial for sustainable forest development, energy flow, and nutrient cycling. However, the dynamics of the biomass, diversity, and species composition of understory vegetation in plantations in south China, along with their key drivers, remain poorly understood. This study investigated four mature plantation types (*Pinus massoniana*, *Pinus caribaea*, *Cunninghamia lanceolata*, and mixed Chinese fir–broadleaf forests) in south China through plot surveys, environmental factor measurements, and structural equation modeling (SEM) to explore the diversity, biomass allocation patterns, and driving mechanisms of understory vegetation. The results demonstrated the following. (1) The introduced Caribbean pine forests exhibited higher shrub biomass than native Masson pine forests, which was driven by their high canopy openness favoring light-demanding species (e.g., *Melicope pteleifolia*, IV = 33.93%), but their low mingling degree limited herb diversity. (2) Masson pine forests showed superior shrub diversity due to their random spatial distribution and higher soil total potassium (TK) content. (3) Mixed Chinese fir–broadleaf forests achieved 24.50–66.06% higher herb biomass compared to coniferous monocultures, supported by high mingling degree, random spatial configuration, and phosphorus-potassium-enriched soil, with concurrently improved herb diversity. SEM revealed that stand structure (DBH, density, mingling degree) directly drove shrub diversity by regulating light availability, while herb biomass was primarily governed by soil total phosphorus (TP) and pH. Canopy-induced light suppression negatively affected herb diversity. We recommend optimizing stand density and canopy structure through thinning and pruning to enhance light heterogeneity alongside supplementing slow-release P fertilizers in P-deficient stands. This study provides theoretical support for the multi-objective management of south China plantations, emphasizing the synergistic necessity of stand structure optimization and soil amendment.

## 1. Introduction

The conservation and restoration of forest ecosystems constitute a critical objective of sustainable development [1,2]. In recent years, this goal has progressively shifted from forest area expansion to biodiversity enhancement [3,4]. Understory vegetation plays a vital role in forest ecosystems. Although its biomass is typically far smaller than that of the canopy layer, its rapid biomass turnover rate exerts significant impacts on ecosystem nutrient cycling, litter decomposition, and microclimate regulation [5]. Furthermore, understory vegetation often represents the primary component of forest biodiversity [6], particularly in plantations, where it contributes substantially to nutrient cycling and energy flow [7]. Consequently, investigating the variations in plantation biodiversity and their underlying drivers has emerged as a focal research topic in recent years [8,9].

In the 1970s, rapid economic growth and infrastructure development in south China led to the extensive degradation of zonal vegetation—primarily evergreen broad-leaved forests [10]. To restore vegetation and improve soil quality, large-scale afforestation programs were initiated, establishing monoculture plantations (e.g., *Pinus massoniana*, *Cunninghamia lanceolata*) and coniferous—broadleaf mixed plantations [11]. However, most plantations in this region have now reached maturity and face multiple ecological challenges, including impeded natural succession [12], soil degradation [13], and unbalanced stand structure [14], collectively driving vegetation diversity decline and compromised ecological functionality. Consequently, there is an urgent need to quantitatively assess understory vegetation diversity and biomass in south China’s plantations, identify key drivers of understory development, and formulate adaptive management strategies to address these issues.

Forest structure and understory light environment are critical determinants of understory vegetation diversity and biomass [15]. Regulating stand density, species composition, and spatial configuration can optimize understory growth conditions, thereby enhancing both biodiversity and biomass accumulation [16]. Empirical studies demonstrate that understory species richness increases with stand density until reaching a threshold, beyond which intensified competition for resources (e.g., light, water, nutrients) suppresses understory vegetation development and reduces diversity [17]. Canopy structural heterogeneity further modulates understory light regimes, influencing the photomorphogenic responses and light-foraging strategies of understory plants [18]. Additionally, tree distribution patterns govern light penetration through canopy gaps, directly affecting the photosynthetic efficiency and growth dynamics of understory communities. Helbach et al. [18] demonstrated that appropriate canopy gap size critically regulates understory plant communities by enhancing light availability, thereby driving species diversity and biomass dynamics.

South China’s subtropical monsoon climate, characterized by high temperatures and abundant rainfall, drives the widespread distribution of acidic lateritic red soils and latosolic red soils (pH < 5.5) with severe nutrient leaching [19]. The interplay between these edaphic properties and vegetation dynamics critically governs the sustainability of plantation ecosystems [20]. Studies confirm that stand type significantly modulates soil nutrient profiles, including total nitrogen (TN), TP, and soil organic carbon (SOC) [21]. For instance, introducing broadleaf species into *Cunninghamia lanceolata* plantations alters the Acidobacteria subpopulation composition and upregulates N-cycling gene abundance, thereby enhancing soil fertility [22]. Furthermore, interspecific variations in litter decomposition rates and nutrient release patterns create divergent soil nutrient availabilities, ultimately shaping understory vegetation composition and productivity [23]. Collectively, stand type mediates understory diversity and biomass through synergistic modifications of soil physicochemical properties and canopy structure.

Despite this progress, the mechanisms underlying these interactions in subtropical plantations remain poorly resolved. Critical knowledge gaps persist regarding (1) the interactive effects of multiple drivers (e.g., light × nutrient × stand structure) and (2) their quantitative contributions to understory biomass allocation. For example, while broadleaf admixtures in conifer monocultures may elevate soil P availability via diversified litter inputs [24], whether this facilitation significantly enhances understory growth compared to conifer monocultures remains unverified. Moreover, no comparative studies have addressed the understory characteristics between exotic species (e.g., *Pinus caribaea*) and native counterparts (e.g., *Pinus massoniana*), leaving their ecological equivalence in plantation management unresolved.

Building upon this foundation, we conducted a comparative study across four dominant plantation types in south China: *Pinus massoniana* (native), *Pinus caribaea* (exotic), *Cunninghamia lanceolata* monoculture, and *Cunninghamia lanceolata*–broadleaf mixed forests. Integrating stand structural inventories, understory light environment monitoring, and soil nutrient quantification, we addressed three pivotal questions: (1) Do exotic *Pinus caribaea* plantations exhibit significantly divergent understory vegetation diversity and biomass allocation patterns compared to native *Pinus massoniana* stands under comparable site conditions? (2) Does introducing broadleaf species into *Cunninghamia lanceolata* monocultures enhance understory species richness and biomass productivity through improved light heterogeneity and soil P availability? (3) What synergistic interactions exist among stand structure, light regimes, and soil properties in driving understory vegetation dynamics? To resolve these questions, we employed SEM to disentangle direct and indirect drivers of understory community assembly. Our findings elucidate species-specific adaptation strategies across plantation types, particularly highlighting how exotic conifers trade off understory biodiversity for faster biomass accumulation. This work establishes a quantitative framework for optimizing stand structure–soil–light interactions, informing precision forest management strategies to enhance ecological functions (e.g., carbon sequestration, nutrient cycling) in subtropical plantations while supporting carbon neutrality goals.

## 2. Results

### 2.1. Stand Structure, Light Environmental and Physical and Chemical Properties of the Soil

The four plantation types exhibited distinct differences in stand structure. PMP and PCP monocultures exhibited superior dimensional traits, with DBH, tree height, and crown width measuring 1.30–1.63-fold and 1.30–1.46-fold greater than those in CMP, respectively. Spatial analysis revealed distinct distribution patterns: CMP approached random spatial arrangement (uniform angle index = 0.52), whereas PCP and PMP exhibited aggregated distributions. Species mingling intensity followed the order CMP (0.63) > PMP > PCP > CLP (near-zero) with CLP showing minimal species heterogeneity (Table 1).

Canopy openness and understory light availability varied substantially across stands. CLP and PCP demonstrated 25.6–34.8% greater canopy openness than other types, while PCP and CMP exhibited 107.52–117.69% lower leaf area indices. Consequently, the total light transmission in PCP and CLP exceeded that of PMP and CMP by 38.4–55.2% with diffuse and direct light components showing parallel trends (Table 2).

Edaphic analyses identified pronounced nutrient gradients. PMP soils showed the lowest bulk density (1.19 g·cm^−3^), while PCP contained the highest TN (0.97 g·kg^−1^). Soil pH in CMP (4.47) significantly surpassed PMP (4.06) and PCP (3.97). TP and TK concentrations followed the hierarchy: CMP > CLP > PMP > PCP, highlighting the nutrient-enhancing effect of mixed-species stands (Table 3).

### 2.2. Species Diversity and Species Composition in the Vegetation

Stand types exerted significant differential effects on understory species diversity (Table 4). In the shrub layer, PMP exhibited significantly higher Simpson and Shannon–Wiener indices than CLP. The Margalef index in PMP and PCP was 16.44% and 23.61% greater than in CLP and CMP, respectively. No significant inter-stand differences emerged in the Pielou index.

In the herbaceous layer, the Shannon–Wiener and Pielou indices of the coniferous-broadleaf mixed forest (CMP) were significantly higher than those of the pure coniferous forests (PMP and PCP). Specifically, the Shannon–Wiener index of CMP increased significantly by 31.1% and 25.0% compared to PMP and PCP, respectively. The Pielou index of CMP also showed a significant increase of 56.3% and 31.6% compared to PMP and PCP, respectively (Table 5). This indicates that mixed forest management can lead to higher species evenness in the herbaceous layer.

Stand types exerted distinct influences on understory floristic composition across vegetation strata. In shrub communities, PMP were dominated by *Castanopsis fissa*, *Csychotria asiatica*, *Rhaphiolepis indica,* and *Uvaria macrophylla*, collectively accounting for over 78% of total IV. PCP exhibited a prevalence of *Melicope pteleifolia*, *Ilex asprella*, and *Wendlandia uvariifolia* (IV range: 15.6–24.3%), while CLP showed a high abundance of *Mussaenda pubescens*, *Ficus hirta*, and *Heptapleurum heptaphyllum*. Notably, *H. heptaphyllum* dominated shrub layers in CMP with IV reaching 60.39% (Table S1).

Herbaceous communities demonstrated stronger homogeneity, with *Blechnopsis orientalis* maintaining dominance across all stands (IV > 47.60%). Stand-specific patterns emerged: PMP exhibited an elevated IV of *Adiantum flabellulatum* (28.7%) and *Cibotium barometz* (19.3%), whereas PCP showed a peak IV of A. flabellulatum (36.85%). In CLP, *Pteris semipinnata* (23.56%) co-dominated with *A. flabellulatum*, while CMP displayed secondary dominance by *M. pubescens* (22.12%), with other herb species remaining below 20% IV (Table S2).

### 2.3. Biomass Allocation of Plant Species

**Figure 1:**
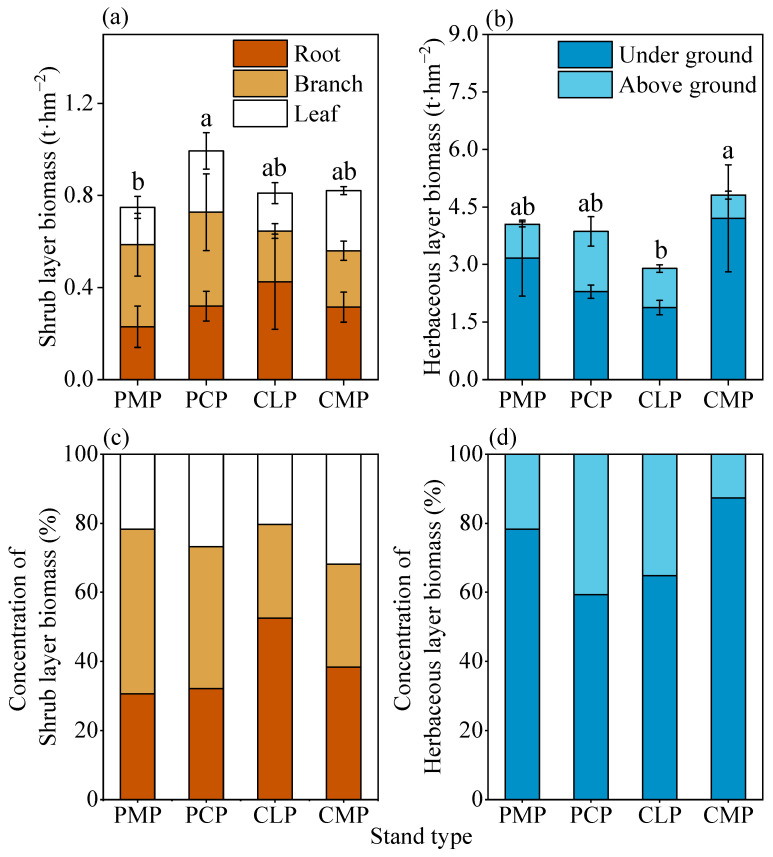
(**a**) Shrub layer biomass in different forest types. (**b**) Herbaceous layer biomass in different forest types. (**c**) Concentration of Shrub layer biomass in different forest types. (**d**) Concentration of Herbaceous layer biomass in different forest types. The distribution and proportion of biomass in the shrub layer and the herb layer. Different lowercase letters indicate significant differences in the total biomass of shrub or herb layers of different stand types (*p* < 0.05). The error bar represents SE (Standard Error, 95% confidence interval).

Shrub layer biomass showed no significant variation across stand types (Figure 1a,c). While PCP exhibited the highest shrub biomass (0.99 t·ha^−1^), followed by CLP (0.82 t·ha^−1^) and CMP (0.81 t·ha^−1^), PMP recorded the lowest (0.75 t·ha^−1^). Biomass allocation patterns diverged markedly: PMP and PCP allocated 47.61% and 41.09% of shrub biomass to branches, whereas CLP and CMP prioritized belowground investment (52.48% and 38.31% root biomass, respectively).

Herbaceous biomass displayed contrasting dynamics with CMP achieving 24.50–66.06% greater than other stands (*p* < 0.05). Belowground biomass dominated across all stands (>59.30% of total), peaking at 72.4% in CLP. Notably, CMP’s herbaceous biomass significantly surpassed CLP by 34.2%, highlighting mixed stands’ capacity for understory productivity enhancement (Figure 1b,d).

### 2.4. The Key Drivers of Understory Vegetation Diversity and Biomass

**Figure 2:**
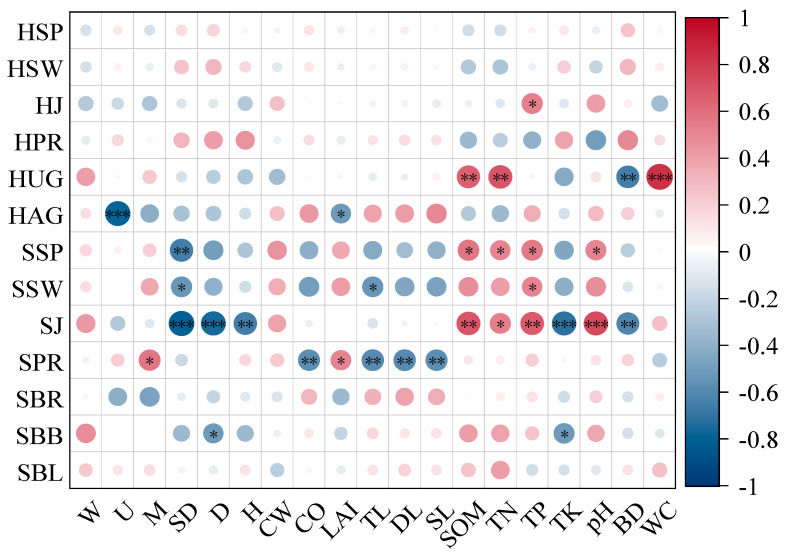
The correlation between the growth indicators of understory vegetation and the driving factors, *: *p* < 0.05, *: *p* < 0.01, *: *p* < 0.001. W: Uniform angle index, U: Dominance ratio, M: Mingling index, SD: Stem density (stems·ha^−1^), D: Mean diameter at breast height (DBH, cm), H: Mean tree height (m), CW: Mean crown width (m), CO: Canopy openness (%), LAI: Leaf area index (m^2^·m^−2^), TL: Total light transmittance (mol·m^−2^·d^−1^), DL: Direct light, SL: Diffuse light, SOM: Soil organic matter (g·kg^−1^), TN: Total nitrogen (g·kg^−1^), TP: Total phosphorus (g·kg^−1^), TK: Total potassium (g·kg^−1^), pH: Soil acidity, BD: Bulk density (g·cm^−3^), WC: Water content (%), HSP: Simpson index, HSW: Shannon–Wiener index, HJ: Pielou evenness index, HPR: Patrick richness index, HUG: Belowground biomass (t·ha^−1^), HAG: Aboveground biomass (t·ha^−1^), SSP: Simpson index, SSW: Shannon–Wiener index, SJ: Pielou index, SPR: Patrick index, SBR: Root biomass (t·ha^−1^), SBB: Branch biomass (t·ha^−1^), SBL: Leaf biomass (t·ha^−1^).

Correlation analysis indicated that the biomass of the herbaceous layer was mainly significantly correlated with the SOM content, TN, bulk density and water content in soil factors. The diversity index of the shrub layer was significantly correlated with the mingling index in the stand structure, average diameter at breast height of the stand, tree height, density, crown width, light intensity, crown openness, and leaf area index in the understory light environment, as well as the SOM, TN, TK, pH, and bulk density in soil factors (Figure 2).

**Figure 3:**
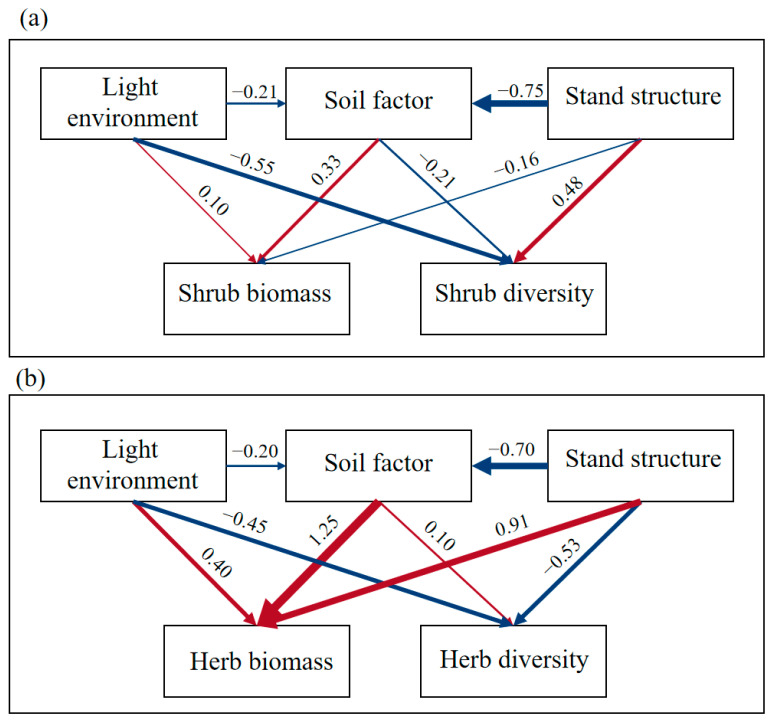
Structural equation model of growth and development and driving factors of understory vegetation. (**a**,**b**) respectively represent the SEM of the growth and development and driving factors of the shrub layer and the herb layer. The red arrows indicate positive relationships and the blue arrows indicate negative relationships. The number next to the arrow is the standardized path coefficient (covariant coefficient), which is proportional to the thickness of the line.

SEM elucidated distinct regulatory pathways. For shrubs (*R*^2^ = 0.745), stand structure directly enhanced diversity (*β* = 0.48) but reduced biomass (*β* = −0.16), while light environment and soil factors showed inverse effects: suppressing diversity (*β* = −0.55 and −0.21) while increasing biomass (*β* = 0.10 and 0.33). Stand characteristics accounted for 62.6% of diversity variation and 62.2% of biomass variation (Figure 3a and Table 6).

The herb layer SEM (*R*^2^ = 0.694) revealed stronger soil mediation: light (*β* = 0.40) and soil factors (*β* = 0.10) positively influenced diversity, contrasting with the stand structure’s suppressive effect (*β* = −0.52). All drivers enhanced herb biomass, particularly soil factors (*β* = 1.25), which exerted 2.8-fold stronger effects than stand structure (*β* = 0.91). Total effect analysis identified soil as the primary herb biomass driver (*β* = 1.25) versus stand structure’s dominance in diversity regulation (*β* = −0.59) (Figure 3b and Table 7).

## 3. Discussion

Understory vegetation, as a crucial component of forest ecosystems, plays an indispensable role in plantations due to its rapid nutrient accumulation and biological turnover rates [5]. However, influenced by species diversity, stand structure, and understory light conditions, vegetation in different stands demonstrates significant spatial heterogeneity in a niche occupation, which is manifested through distinct community composition, vertical stratification, and distribution patterns [25]. As dominant plantation types in south China, *Pinus massoniana* and *Pinus caribaea* stands exhibit marked differences in understory characteristics. *Pinus massoniana* forests generally support higher understory diversity, where naturally regenerated species like *Castanopsis fissa* and *Psychotria asiatica* form stratified shrub communities resembling early successional features of tropical secondary forests. This phenomenon likely stems from complex interdependencies with local soil, climate, and biotic factors, with long-term co-evolution enabling *Pinus massoniana* stands to sustain richer native understory communities [26]. In contrast, *Pinus caribaea* stands with greater canopy openness, favoring light-demanding species such as *Melicope pteleifolia*, *Ilex asprella*, and *Wendlandia uvariifolia* that dominate ecological niches while suppressing other vegetation [27]. This intensified light availability concurrently contributes to greater shrub biomass accumulation in *Pinus caribaea* stands compared to *Pinus massoniana* plantations.

In comparison, the Chinese fir–broadleaf mixed forest’s high density (2016 trees·hm^−2^) and random distribution pattern (angular scale 0.52) likely suppress shrub growth through root competition and light interception, while its elevated species mingling degree (0.63) enhances stand habitat heterogeneity, thereby promoting herbaceous layer biomass accumulation. The introduction of broadleaf species into pure stands may accelerate nutrient cycling through diversified litter composition and quality differences [28], consequently improving herb layer diversity. While understory light resources provide essential energy for plant growth, they may simultaneously inhibit shade-tolerant species [29], potentially explaining the lower herb diversity in Chinese fir pure stands compared to mixed forests. The mixed forest’s reduced light intensity appears to drive herbaceous plants toward photo adaptive strategies, such as increased leaf area and thicker laminar structures, resulting in 24.5–66.1% greater herb biomass than pure Chinese fir stands. These physiological adaptations likely correlate with species-specific functional traits (e.g., specific leaf area, chlorophyll concentration), though experimental validation remains necessary [30]. Furthermore, beyond the live canopy (LAI), other factors likely influenced the understory light environment. We observed that *Cunninghamia lanceolata* forest monocultures often had a dense, persistent layer of undecomposed *Cunninghamia lanceolata* needles, which could create a physical barrier and further reduce light penetration to the herbaceous layer [31]. In contrast, the mixed stands had a more heterogeneous litter layer composed of broadleaf and conifer litter, which likely decomposed faster and formed a less continuous barrier. The retention of dead lower branches, particularly in the dense *Cunninghamia lanceolata* stands, may have contributed to the interception of light before it reached the forest floor, a factor not captured by our hemispherical photography which primarily assesses the live canopy.

Soil physicochemical properties significantly influence understory vegetation’s resource allocation strategies, particularly determining the distribution patterns of shallow-rooted herbs and shrubs [31]. P and K play crucial roles in plant energy metabolism (ATP synthesis) and water regulation (stomatal control). Although P deficiency commonly occurs in south China’s lateritic red soils [32], the Chinese fir–broadleaf mixed forest exhibits superior TP (0.83 g·kg^−1^) and TK (33.65 g·kg^−1^) concentrations, potentially explaining its exceptional herbaceous biomass (4.05 t·hm^−2^). This nutrient enrichment likely stems from diversified litter inputs—broadleaf species like *Cinnamomum camphora* contribute a low C/N ratio litter that accelerates microbial mineralization [33], while reduced bulk density (1.26 g·cm^−3^) facilitates root proliferation and nutrient uptake efficiency. Notably, Caribbean pine stands demonstrate superior carbon sequestration capacity with SOM content 1.64-fold higher than Masson pine forests, coupled with elevated moisture levels mitigating seasonal drought stress. However, concerning evidence suggests Caribbean pine litter contains elevated resin acids and other allelopathic compounds that may inhibit P mineralization through microbial suppression [34], potentially causing long-term P depletion that compromises understory productivity, which particularly affects shallow-rooted herbs dependent on labile nutrient pools [35].

In biomass allocation, shrubs in Chinese fir pure stands and fir–broadleaf mixed forests preferentially allocate greater proportions to root systems (52.48% and 38.31% respectively), while *Pinus massoniana* and *Pinus caribaea* stands exhibit predominant biomass investment in aboveground branches (47.61% and 41.09%). This divergence likely relates to soil resource heterogeneity—Chinese fir stands with the highest soil bulk density (1.41 g·cm^−3^) may mechanically force shrubs to develop deeper root systems for water acquisition [36], whereas elevated organic matter content in *Pinus massoniana* and *Pinus caribaea* stands could reduce root construction costs, possibly reducing belowground investment while promoting aerial photic competition [37].

SEM analysis revealed that stand structure exerted significantly stronger direct positive effects on shrub diversity (path coefficient = 0.48) than the negative impacts from light environment and soil factors, confirming the critical role of canopy management practices (e.g., thinning and pruning) in regulating understory vegetation [6]. The herbaceous layer biomass showed a primary dependence on soil factors (path coefficient = 1.25), particularly TP and pH regulation, suggesting that P fertilizer application or soil amendments could effectively enhance herb productivity in plantation management. Notably, contrasting responses emerged between vegetation layers—the understory light environment positively influenced herb diversity (path coefficient = 0.40) while negatively affecting shrubs (path coefficient = −0.55), reflecting niche differentiation in photic adaptation across plant life forms [38].

It is important to note that the quantitative strengths of the drivers identified (e.g., the primacy of soil P for herb biomass) may vary across different geographical locations and soil types in south China’s extensive plantation landscapes. Therefore, our findings should be interpreted as a mechanistic case study that reveals key interactive processes. The management suggestions below are proposed as testable hypotheses for local application, which should be validated through broader regional studies before large-scale implementation. (1) Structural optimization of Chinese fir monocultures: Implement the gradual introduction of shade-tolerant broadleaf species (e.g., *Cinnamomum burmannii*, *Schima superba*) to establish coniferous–broadleaf mosaic structures. (2) Nutrient management in pine-dominated stands: Address P limitations through slow-release P fertilizers during tending phases or enhanced nutrient cycling via litter retention. This study provides scientific basis for ecological restoration and the sustainable management of subtropical plantations, though long-term monitoring remains essential to decipher dynamic responses of understory vegetation to climate change and anthropogenic disturbances. Future research should prioritize investigating soil microbiome–vegetation interactions across stand types and climate change impacts on understory community assembly.

## 4. Materials and Methods

### 4.1. Overview of the Research Area

The study area was located in Yunyong Forest Farm (112°38′26″–112°42′25″ E, 22°41′54″–22°46′50″ N) in Foshan City, Guangdong Province, which is characterized by a humid subtropical monsoon climate with abundant rainfall. The region exhibits a mean annual temperature of 23.2 °C and annual precipitation of approximately 1900 mm. Soils are predominantly lateritic red soils derived from granite and sandy shale weathering, with a soil layer thickness of 0.4–1.0 m and humus layer of 2.5–4.0 cm, displaying acidic properties (pH < 5.2). Dominant forest types include subtropical evergreen broad-leaved forests, mixed conifer–broadleaf stands, and coniferous monocultures. The investigated forest stands were respectively a 31-year-old *Pinus caribaea* forest, a 28-year-old *Pinus massoniana* forest, a 26-year-old *Cunninghamia lanceolata* forest and a 26-year-old mixed conifer–broadleaf stands (the mixed conifer-broadleaf stands were established with Chinese fir interplanted with native broadleaf species (*Camphora officinarum*, *Cinnamomum burmanni* etc.) in a non-uniform, mixed planting pattern, resulting in a stand with a high mingling degree; see Table S1). In the first three years of the four forest stands, mowing, weeding and fertilization were carried out, and no tending measures were taken from the fourth to the seventh year. In the 8th year, light cutting and pruning were carried out, and in the 10th and 11th years, thinning and tending were performed.

### 4.2. Plot Setting

**Figure 4:**
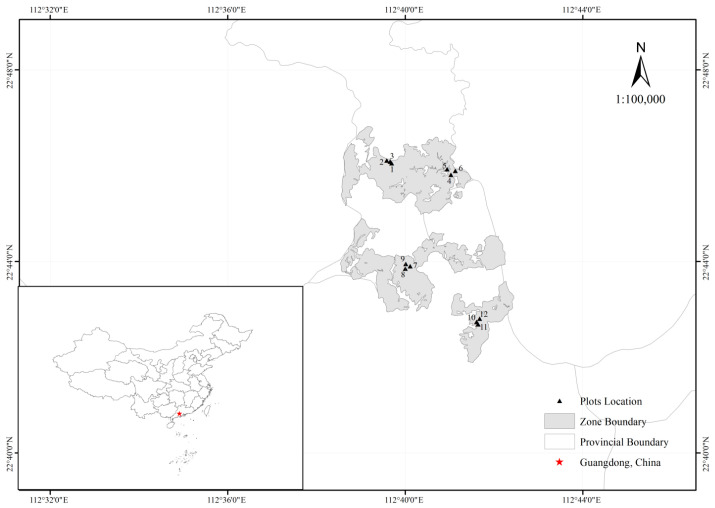
General characteristics of sample plots in subtropical plantations.

**Figure 5:**
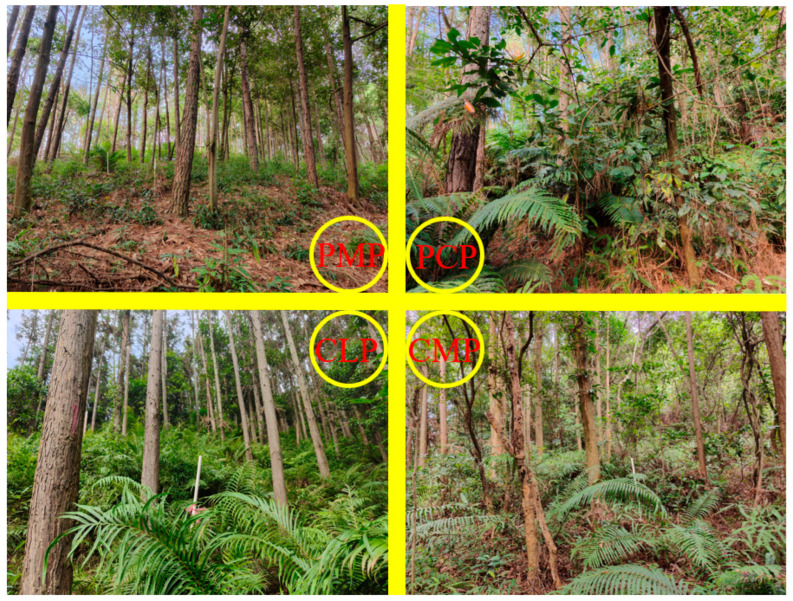
Four types of forest stand within the study area.

Four stand types (*Pinus massoniana*, *Pinus caribaea*, *Cunninghamia lanceolata*, and mixed conifer–broadleaf stands) with comparable site conditions were selected within the study area. Three replicate plots (20 × 20 m) per stand type (total 12 plots) were systematically established following a randomized block design. To ensure statistical independence between stands, a minimum 15 m buffer zone was maintained between adjacent plots. Field investigations were conducted during April–May 2023, corresponding to the early rainy season (April–June) in subtropical south China. This timing capitalizes on peak vegetation phenophases, ensuring comprehensive species representation during active growth periods while minimizing seasonal sampling artifacts [39]. Understory vegetation surveys included woody regeneration cohorts with saplings (height < 0.5 m) explicitly classified as understory components. Multivariate analyses incorporated stand structural parameters and soil nutrients to holistically evaluate drivers of understory vegetation dynamics. The geospatial distribution of sampling plots is illustrated in Figure 4 with key stand characteristics summarized in Table S1 and Figure 5.

### 4.3. Indicator Calculation

#### 4.3.1. Forest Stand Structure

Stand structural parameters were categorized into spatial and non-spatial attributes. This study focused on four non-spatial structural metrics: mean stand diameter at breast height (D, cm), mean tree height (H, m), mean crown width (CW, m), and stem density (SD, trees·ha^−1^). During April–June 2023, field measurements were systematically conducted: DBH was measured using diameter tapes at 1.3 m height, H was determined via laser hypsometer for all individuals with D ≥ 5 cm, and crown dimensions were recorded through orthogonal measurements using fiberglass tapes. Stem density was calculated from tree counts per 20 m × 20 m plot, which were scaled to a hectare basis.

**Figure 6:**
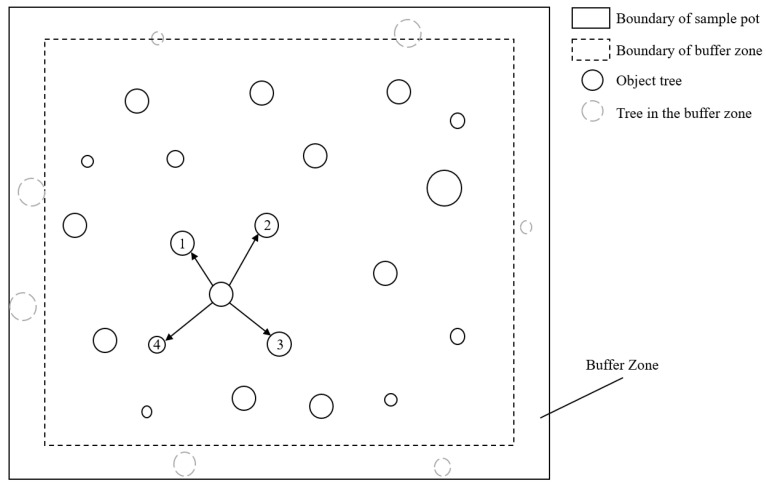
Unit schematic diagram of the spatial structure of the forest stand. The adjacent trees *n* = 4, and the buffer zone = 5 m.

Three key spatial structural indices were analyzed: the uniform angle index (W) quantifies angular distribution regularity of nearest neighbors [40]; the dominance ratio (U) assesses size hierarchy relative to adjacent trees [40]; and the mingling index (M) measures species spatial heterogeneity [41]. Computation followed the structural unit approach, where each subject tree and its 4 nearest neighbors (n = 4) constituted an analytical unit. To mitigate edge effects, a 5 m buffer zone was established along plot boundaries, subject trees were prohibited within buffer areas, and neighbor trees outside plots were excluded from calculation (Figure 6). Mathematical formulations and ecological interpretations are provided below:

(1) $$W_{i}=1/n\sum_{j=1}^{4}Z_{ij}$$

(2) $$U_{i}=1/N_{1}\sum_{n_{t}}^{1}K_{ij}$$

(3) $$M_{i}=\frac{1}{4}\;\sum_{j=1}^{4}v_{ij}$$

where $Z_{ij}$ is a binary variable: $Z_{ij}$ = 1 if the jth adjacent tree’s azimuth angle exceeds the reference angle α_0_ (72°); otherwise $Z_{ij}$ = 0. $K_{ij}$ is an indicator variable: $K_{ij}$ = 1 if the subject tree i has a smaller D than neighboring tree j; otherwise $K_{ij}$ = 0. $v_{ij}$ is a species dissimilarity index: $v_{ij}$ = 1 if neighboring tree j belongs to a different species than subject tree i; otherwise, $v_{ij}$ = 0.

#### 4.3.2. Light Environment

Hemispherical canopy photographs were acquired using a Canon EOS 50D Mark II camera (Canon Inc., Ota City, Japan) equipped with an 8–15 mm f/4 L fisheye lens (Canon Inc., Japan). At each plot, three randomly selected sampling points (>5 m apart) were photographed vertically upward from 50 cm above ground level between 09:00–10:00 daily under clear-sky conditions. These images were subsequently analyzed in Gap Light Analyzer (GLA) v2.0 [42] to quantify five understory light parameters: leaf area index (LAI, m^2^·m^−2^), canopy openness (CO, %), total light transmittance (TL, mol·m^−2^·d^−1^), diffuse light (SL, mol·m^−2^·d^−1^), and direct light (DL, mol·m^−2^·d^−1^).

#### 4.3.3. Physical and Chemical Properties of Soil

Soil samples were collected using a combination of composite sampling for chemical analysis and core sampling for physical analysis. For soil chemical properties (SOC, TN, TP, TK, pH), a composite sampling strategy was employed. Within each plot, five soil subsamples were randomly collected from the 0–20 cm depth layer and thoroughly mixed to form one composite sample per plot per depth in order to capture the plot-level heterogeneity. For soil physical properties (bulk density, water content), three replicate undisturbed core samples were taken per plot at the 0–20 cm depth using 8 cm diameter stainless steel core cutters, maintaining a minimum 5 m spacing between sampling points. All analyses were performed on these composite (chemistry) or replicated (physics) samples.

For the remaining parameters, we used the following analytical methods: SOC: K dichromate oxidation [43], TN and TP: elemental analyzer (Vario EL cube, Elementar, Langenselbold, Germany), TK: flame photometry (FP-640, INESA, Shanghai, China), pH: digital pH meter (P5S-2F, LEICI, Shanghai, China) calibrated with standard buffers [44], bulk density: Core cutter method via oven—drying at 105 °C to constant mass [45].

#### 4.3.4. Diversity, Important Values and Biomass of Vegetation

To quantify understory vegetation diversity, five 2 × 2 m shrub quadrats were systematically established per plot (four corners + center). Within each shrub quadrat, a nested 1 × 1 m herbaceous subplot was positioned at the southwest corner. All vascular plant species within quadrats were taxonomically identified to species level. Four diversity indices were calculated: Simpson dominance index (SP), Shannon–Wiener diversity index (SW), Margalef richness index (PR), and Pielou evenness index (*J*) [46,47].

For both shrub and herbaceous layers, the aboveground and belowground biomass were determined via destructive harvesting within the quadrats and subplots, respectively. After clipping the aboveground parts, the belowground biomass (roots) was carefully excavated. For shrubs, the root systems of the entire individual within the quadrat were excavated. For herbs, all roots within the top 20 cm of soil in the subplot were collected. During excavation, we meticulously distinguished the fine and coarse roots of the target understory individuals from those of overstorey trees based on morphological differences (e.g., color, texture, diameter, and branching patterns) and by tracing the roots back to the target plant. Only roots that could be unequivocally linked to a sampled understory individual were collected. All biomass samples were oven-dried at 60 °C to constant mass (±0.01 g precision) and converted to dry biomass equivalents [48].

The importance value (*IV*) was a synthetic metric reflecting the species’ ecological dominance. This composite integrated index elucidates species-specific roles in community assembly and resource partitioning [49,50], which was calculated as

(4) IV=(RA+RF+RC)/3

The Simpson dominance index quantifies the probability that two randomly selected individuals belong to the same species. Contrary to intuition, higher values indicate lower diversity, which was calculated as

(5) $$SP=1-\sum_{i=1}^{S}P_{i}^{2}$$

The Shannon–Wiener diversity index measures community heterogeneity through species’ proportional abundances:

(6) $$SW=\sum_{i=1}^{S}P_{i}\ln P_{i}$$

The Margalef richness index assesses species number relative to sample size:

(7) PR=H/lnS

The Pielou evenness index evaluates the equitability of species abundance distribution:

(8) $$R=\frac{S-1}{\ln N}$$

where IV represents the important value of the shrub/herb layers; and RA stands for relative abundance, representing the number of a certain species divided by the total number of all species. RF is the relative frequency, representing the ratio of the frequency of a certain species in all quadrat plots to the total frequency of all species. RC stands for relative dominance, representing the ratio of the coverage of a certain species to the aspect volume of that sample. S represents the number of species in the community, N represents the total number of observed individuals, and $P_{i}$ represents the proportion of the number of individuals of species *i* to the total number of individuals in the community.

#### 4.3.5. Data Processing

Statistical analyses were conducted using SPSS 19.0 (SPSS Inc., Chicago, IL, USA) and graphical visualizations created in Origin Pro 2019 (Origin Lab Corporation, Northampton, MA, USA). Significant differences among the four stand types (PMP, PCP, CLP, CMP) for each individual dependent variable (e.g., DBH, diversity indices, soil nutrients) were assessed through one-way analysis of variance (ANOVA) with a significance level of *α* = 0.05. Post hoc comparisons were performed using Tukey’s Honest Significant Difference (HSD) test (*α* = 0.05). Bivariate relationships between drivers and understory vegetation parameters were examined via Pearson correlation analysis. Structural Equation Modeling (SEM) was implemented in Smart PLS v4.1.0.3 (Smart PLS GmbH, Bönningstedt, Germany) to disentangle direct/indirect effects among five latent constructs: stand structure (mean DBH, tree height, crown width, uniform angle index, dominance ratio, mingling index, stem density), understory light regime(canopy openness, leaf area index, total/diffuse/direct light), edaphic factors (pH, SOM, TN, TP, TK), moisture content, bulk density, shrub layer dynamics (diversity indices and biomass), herb layer dynamics (diversity indices and biomass). The hypothesized path model evaluated 23 measurement variables across these constructs. Model fit was assessed using bootstrap resampling (5000 iterations) with significance threshold *p* < 0.05. All data are presented as mean ± standard deviation (SD).

## 5. Conclusions

This study systematically investigated the understory vegetation diversity and biomass characteristics across four subtropical plantations in south China, elucidating their driving mechanisms. Key findings include the following:

(1) Native vs. exotic species contrast: *Pinus massoniana* stands exhibited higher shrub diversity (dominated by *Castanopsis fissa*) due to their larger diameter at DBH, broader crown widths, and random spatial patterns. In contrast, *Pinus caribaea* stands demonstrated greater shrub biomass (dominated by *Melicope pteleifolia* and *Wendlandia uvariifolia*), which was attributed to their superior soil organic matter and moisture content. (2) Monoculture vs. mixed plantation comparison: The Chinese fir–broadleaf mixed forest displayed 24.50–66.06% higher herbaceous biomass and enhanced diversity, which was driven by their high mingling degree, random distribution patterns, and enriched phosphorus–potassium soil conditions. (3) Differentiated drivers: Stand structure parameters (D, density, mingling degree) directly regulated shrub development through spatial configuration and light redistribution (path coefficient = 0.48), while herbaceous layers were predominantly controlled by soil–light interactions, particularly P availability and pH modulation.

These findings reveal the adaptive strategies of understory vegetation to heterogeneous environments in subtropical plantations, highlighting critical constraints from inappropriate stand structures and P-deficient acidic soils. Our structural equation models revealed that stand structure is a key driver of understory dynamics. This finding suggests that manipulating stand density through thinning could be a potential management tool to improve light availability and heterogeneity. However, the optimal intensity and timing of thinning remain unknown. Therefore, we recommend that future research prioritizes long-term thinning experiments that monitor the response of understory vegetation and soil biota to a gradient of stocking densities. Such manipulative studies are essential to move from correlative understanding to predictive, prescriptive management. This work advances our understanding of understory ecological processes in subtropical plantations, offering actionable insights for sustainable forest management and carbon-neutral initiatives.

## Figures and Tables

**Table 1:** The forest structure of different stand types.

Index	Stand Type			
	PMP	PCP	CLP	CMP
Uniform angle	0.55 ± 0.00 ab	0.58 ± 0.01 a	0.53 ± 0.02 ab	0.52 ± 0.02 b
Neighborhood comparison	0.49 ± 0.02 a	0.50 ± 0.00 a	0.50 ± 0.01 a	0.50 ± 0.00 a
Mingling degree	0.31 ± 0.06 b	0.22 ± 0.08 b	0.05 ± 0.01 c	0.63 ± 0.03 a
Stand density (tree·hm^−2^)	891 ± 44 b	808 ± 156 b	1208 ± 100 ab	2016 ± 186 a
Mean DBH (cm)	22.23 ± 0.62 a	19.86 ± 1.10 ab	17.31 ± 0.35 b	13.63 ± 0.26 c
Mean tree height (m)	18.22 ± 1.06 a	16.93 ± 1.11 ab	15.48 ± 0.35 b	11.77 ± 0.16 c
Mean crown width (m)	5.76 ± 0.41 a	5.54 ± 0.32 a	3.59 ± 0.17 b	4.25 ± 0.21 b

Note: PMP stands for pure *Pinus massoniana*, PCP stands for pure *Pinus caribaea*, CLP stands for pure *Cunninghamia lanceolata*, and CMP stands for mixed fir and broadleaved forest. Data presented as mean ± standard error (SE); *n* = 3. Within rows, means followed by different lowercase letters differ significantly according to Tukey’s multiple range test (*p* < 0.05).

**Table 2:** Understory light environment of different sand types.

Index	Stand Type			
	PMP	PCP	CLP	CMP
Canopy openness (%)	10.44 ± 1.03 b	31.53 ± 2.57 a	31.29 ± 3.06 a	10.06 ± 0.69 b
Leaf area index (cm^2^·cm^−3^)	2.76 ± 0.17 a	1.30 ± 0.10 b	2.83 ± 0.10 a	1.33 ± 0.15 b
Total light (cd·m^−2^)	3.43 ± 0.47 b	10.5 ± 0.38 a	11.45 ± 0.98 a	3.58 ± 0.44 b
Direct light (cd·m^−2^)	1.61 ± 0.34 b	5.09 ± 0.22 a	6.02 ± 0.63 a	1.84 ± 0.27 b
Scattered light (cd·m^−2^)	1.82 ± 0.20 b	5.42 ± 0.29 a	5.42 ± 0.40 a	1.74 ± 0.20 b

Note: PMP stands for pure *Pinus massoniana*, PCP stands for pure *Pinus caribaea*, CLP stands for pure *Cunninghamia lanceolata*, and CMP stands for mixed fir and broadleaved forest. Data presented as mean ± standard error (SE); *n* = 3. Within rows, means followed by different lowercase letters differ significantly according to Tukey’s multiple range test (*p* < 0.05).

**Table 3:** Soil physicochemical properties of different stand types.

Index	Stand Type			
	PMP	PCP	CLP	CMP
Organic matter (g.kg^−1^)	16.96 ± 1.02 b	27.91 ± 1.16 a	10.84 ± 1.76 c	14.09 ± 0.69 bc
Total N (g.kg^−1^)	0.65 ± 0.03 b	0.97 ± 0.04 a	0.48 ± 0.03 b	0.62 ± 0.02 b
Total P (g.kg^−1^)	0.21 ± 0.01 b	0.18 ± 0.01 b	0.24 ± 0.01 b	0.83 ± 0.24 a
Total K (g.kg^−1^)	16.74 ± 2.37 b	7.26 ± 0.61 c	32.36 ± 1.81 a	33.65 ± 4.37 a
pH	4.06 ± 0.04 b	3.97 ± 0.03 b	4.26 ± 0.05 ab	4.47 ± 0.07 a
Bulk density (g·cm^−3^)	1.36 ± 0.02 a	1.19 ± 0.05 b	1.41 ± 0.05 a	1.26 ± 0.04 ab
Water content (%)	24.79 ± 1.61 b	37.94 ± 1.34 a	24.28 ± 0.51 b	26.69 ± 1.56 b

Note: PMP stands for pure *Pinus massoniana*, PCP stands for pure *Pinus caribaea*, CLP stands for pure *Cunninghamia lanceolata*, and CMP stands for mixed fir and broadleaved forest. Data presented as mean ± standard error (SE); *n* = 3. Within rows, means followed by different lowercase letters differ significantly according to Tukey’s multiple range test (*p* < 0.05).

**Table 4:** Diversity in the shrub layer of different forest stands types.

Stand Type	Simpson	Shannon-Wiener	Margalef	Pielou
PMP	0.85 ± 0.01 a	2.32 ± 0.12 a	0.85 ± 0.01 a	15.33 ± 1.76 a
PCP	0.80 ± 0.08 ab	1.91 ± 0.41 ab	0.89 ± 0.06 a	9.33 ± 3.38 b
CLP	0.67 ± 0.02 b	1.54 ± 0.07 b	0.73 ± 0.01 b	8.33 ± 0.88 b
CMP	0.74 ± 0.02 ab	1.78 ± 0.05 ab	0.72 ± 0.03 b	12.33 ± 2.03 ab

Note: PMP stands for pure *Pinus massoniana*, PCP stands for pure *Pinus caribaea*, CLP stands for pure *Cunninghamia lanceolata*, and CMP stands for mixed fir and broadleaved forest. Data presented as mean ± standard error (SE); *n* = 3. Within rows, means followed by different lowercase letters differ significantly according to Tukey’s multiple range test (*p* < 0.05).

**Table 5:** Diversity in the herbaceous layer of different forest stands types.

Stand Type	Simpson	Shannon-Wiener	Margalef	Pielou
PMP	0.73 ± 0.05 a	1.45 ± 0.21 b	0.90 ± 0.03 a	5.33 ± 1.33 b
PCP	0.72 ± 0.07 a	1.52 ± 0.22 b	0.84 ± 0.03 a	6.33 ± 1.20 b
CLP	0.78 ± 0.05 a	1.75 ± 0.11 ab	0.86 ± 0.06 a	7.67 ± 0.33 ab
CMP	0.84 ± 0.07 a	1.90 ± 0.21 a	0.91 ± 0.05 a	8.33 ± 1.20 a

Note: PMP stands for pure *Pinus massoniana*, PCP stands for pure *Pinus caribaea*, CLP stands for pure *Cunninghamia lanceolata*, and CMP stands for mixed fir and broadleaved forest. Data presented as mean ± standard error (SE); *n* = 3. Within rows, means followed by different lowercase letters differ significantly according to Tukey’s multiple range test (*p* < 0.05).

**Table 6:** Path analysis of growth and development indicators and driving factors of shrub layers.

Latent Variable	Observational Variable	Direct Effect	Indirect Effect	Total Effect
Shrub diversity	Light environment	−0.55	0.05	−0.50
	Soil factor	−0.21	/	−0.21
	Stand structure	0.48	0.16	0.64
Shrub biomass	Light environment	0.10	−0.07	0.03
	Soil factor	0.33	/	0.33
	Stand structure	−0.16	−0.25	−0.41

**Table 7:** Path analysis of growth and development indicators and driving factors of the herbaceous layer.

Latent Variable	Observational Variable	Direct Effect	Indirect Effect	Total Effect
Herb diversity	Light environment	0.40	−0.02	0.38
	Soil factor	0.10		0.10
	Stand structure	−0.52	−0.07	−0.59
Herb Biomass	Light environment	0.45	−0.26	0.19
	Soil factor	1.25		1.25
	Stand structure	0.91	−0.87	0.04

## Data Availability

Data will be made available on request.

## Supplementary Materials

The following supporting information can be downloaded at: https://www.mdpi.com/article/10.3390/plants14223452/s1: Table S1: Overview of the 12 sample plots. Table S2: Important values of shrub layers in different forest stand types. Table S3: Important values of herbaceous layers in different forest stand types.
