# Supplementary material for: Adaptation Mechanisms of Understory Vegetation in Subtropical Plantations: Synergistic Drivers of Stand Spatial Structure and Soil Fertility
Source: Plants (Basel). 2025 Nov 11;14(22):3452. doi: 10.3390/plants14223452 (PMC12656622; doi:10.3390/plants14223452)
Supplement: Supplementary file 1 [file plants-14-03452-s001.zip › plants-3939792-supplementary.pdf]

Adaptation Mechanisms of Understory Vegetation in Subtropical Plantations: Synergistic Drivers of Stand Spatial Structure and Soil Fertility

Fenglin Zheng [1], Dehao Lu [1], Wenyi Ou [2], Sha Tan [3], Xiongjian Xu [3], Shucai Zeng [1] and Lihua Xian [1*]

1. College of Forestry & Landscape Architecture, South China Agricultural University, Guangzhou 510640, China

2. College of Horticulture and Landscape Architecture, Zhongkai Agricultural Engineering College, Guangzhou 519080, China

3. Foshan Yunyong Forest Farm, Foshan 528518, China

* Corresponding Author: Lihua Xian

College of Forestry and Landscape Architecture, South China Agricultural University, 483 Wushan Road, Wushan Street, Guangzhou 510642, PR China.

E-mail: xianlihua@scau.edu.cn

Supporting information, including 3 tables.

Table S1. Overview of the 12 sample plots and their dominant canopy tree species

Stand Type	Plots	Dominant Species	Age (a)	Mean DBH (cm)	Mean Tree Height (m)	Tree Density (Trees·ha^{-1})	Canopy density (%)	Elevation (m)	Slope (°)
PMP	1	*Pinus massoniana* Lamb.	28	21.23	16.13	1000	0.90	95	28
	2			21.76	18.97	825	0.91	95	23
	3			23.71	19.56	850	0.88	95	22
PCP	4	*Pinus caribaea* Morelet.	31	17.81	15.5	1175	0.64	194	26
	5			19.38	16.19	725	0.69	191	25
	6			22.39	19.11	825	0.72	198	24
CLP	7	*Cunninghamia lanceolata* (Lamb.) Hook.	26	17.68	12.02	1450	0.74	168	29
	8			17.78	11.61	1125	0.68	184	25
	9			16.46	10.82	1050	0.64	198	27
CMP	10	*Cunninghamia lanceolata* (Lamb.) Hook.	26	13.04	8.46	2450	0.91	105	22
	11	*Cinnamomum burmanni* (Nees & T. Nees) Blume.		13.71	8.84	1675	0.89	110	26
	12	*Camphora officinarum* var., etc.		14.14	9.00	1925	0.90	115	27

Note: PMP stands for *Masson pine* plantation, PCP for *Caribbean pine* plantation, CLP for *Cunninghamia lanceolata* plantation, and CMP for mixed conifer-broadleaf stands. For CMP, all listed species were present as a mixture within each of the three replicate plots.

Table S2. Important values of shrub layers in different forest stand types

Species	Family	Genus	Important value (%)			
			PMP	PCP	CLP	CMP
Litsea glutinosa (Lour.) C. B. Rob.	*Lauraceae*	*Litsea*	11.83			
Ilex asprella (Hook. & Arn.) Champ. ex Benth.	*Aquifoliaceae*	*Ilex*	11.32	23.45		
Ficus hirta Vahl	*Moraceae*	*Ficus*	10.49	17.14	20.01	5.05
Psychotria asiatica L.	*Rubiaceae*	*Psychotria*	24.40		7.09	
Castanopsis fissa (Champ. ex Benth.) Rehder & E.H.Wilson	*Fagaceae*	*Castanopsis*	54.14			
Ardisia quinquegona Blume	*Primulaceae*	*Ardisia*	5.24			
Eurya chinensis R. Br.	*Pentaphylacaceae*	*Eurya*	5.76			
Melicope pteleifolia (Champ. ex Benth.) Hartley	*Rutaceae*	*Melicope*	8.56	33.93	16.57	7.77
Triadica cochinchinensis Lour.	*Euphorbiaceae*	*Triadica*	5.13			
Rhaphiolepis indica (L.) Lindl.	*Rosaceae*	*Rhaphiolepis*	13.83			
Embelia laeta (L.) Mez	*Primulaceae*	*Embelia*	10.93			
Strophanthus divaricatus (Lour.) Hook. & Arn.	*Apocynaceae*	*Strophanthus*	5.34	5.63		
Mussaenda pubescens W. T. Aiton	*Rubiaceae*	*Mussaenda*	7.82	6.03	40.72	13.91
Gardenia jasminoides J. Ellis	*Rubiaceae*	*Gardenia*	6.57			
Uvaria macrophylla Roxb.	*Annonaceae*	*Uvaria*	13.10			
Sarcandra glabra (Thunb.) Nakai	*Chloranthaceae*	*Sarcandra*		6.02		18.18
Symplocos lancifolia Siebold & Zucc.	*Symplocaceae*	*Symplocos*		6.81		
Rubus reflexus var. lanceolobus	*Rosacea*	*Rubus*		5.90	9.08	
Wendlandia uvariifolia Hance	*Rubiaceae*	*Wendlandia*		22.68	12.35	16.17
Maesa perlarius (Lour.) Merr.	*Primulaceae*	*Maesa*	9.63			11.88
Litsea cubeba (Lour.) Pers.	*Lauraceae*	*Litsea*	7.36			
Heptapleurum heptaphyllum (L.) Y. F. Deng	*Araliaceae*	*Heptapleurum*			20.50	60.39
Aporosa dioica (Roxb.) Müll.Arg.	*Phyllanthaceae*	*Aporosa*			10.27	9.27
Alangium chinense (Lour.) Harms	*Cornaceae*	*Alangium*				8.92
Diospyros morrisiana Hance	*Ebenaceae*	*Diospyros*				13.26
Glochidion eriocarpum Champ. ex Benth.	*Phyllanthaceae*	*Glochidion*				5.22
Cinnamomum cassia (L.) J. Presl	*Lauraceae*	*Cinnamomum*				5.12
Cunninghamia lanceolata (Lamb.) Hook.	*Cupressaceae*	*Cunninghamia*				7.96
Tetracera sarmentosa (L.) Vahl	*Dilleniaceae*	*Tetracera*				6.37

Note: Only plant species with *IV* greater than 5% were counted among different stands.

Table S3. Important values of herbaceous layers in different forest stand types

Species	Family	Genus	Important value (%)			
			PMP	PCP	CLP	CMP
Blechnopsis orientalis (L.) C. Presl	*Blechnaceae*	*Blechnopsis*	49.55	47.60	60.00	66.15
Adiantum flabellulatum L.	*Pteridaceae*	*Adiantum*	23.86	5.59	36.85	19.76
Cibotium barometz (L.) J. Sm.	*Cibotiaceae*	*Cibotium*	15.96	38.96	5.98	
Woodwardia japonica (L. f.) Sm.	*Blechnaceae*	*Woodwardia*	12.62		22.79	
Lophatherum gracile Brongn.	*Poaceae*	*Lophatherum*	7.43	9.23		8.93
Oplismenus compositus (L.) P. Beauv.	*Poaceae*	*Oplismenus*	6.52			
Mussaenda pubescens W. T. Aiton	*Rubiaceae*	*Mussaenda*	6.48	5.18		22.12
Dicranopteris pedata (Houtt.) Nakaike	*Gleicheniaceae*	*Dicranopteris*	45.24			
Tectaria subtriphylla (Hook. & Arn.) Copel.	*Tectariaceae*	*Tectaria*		8.23		18.21
Clerodendrum cyrtophyllum Turcz.	*Lamiaceae*	*Clerodendrum*		6.18		
Dioscorea cirrhosa Lour.	*Dioscoreaceae*	*Dioscorea*		5.33		
Pronephrium gymnopteridifrons (Hayata) Holttum	*Thelypteridaceae*	*Pronephrium*		5.10		
Lygodium japonicum (Thunb.) Sw.	*Lygodiaceae*	*Lygodium*		5.01	8.91	
Pteris semipinnata L.	*Pteridaceae*	*Pteris*			23.56	11.43
Nekemias cantoniensis (Hook. & Arn.) J. Wen & Z. L. Nie	*Vitaceae*	*Nekemias*			17.31	
Osmunda japonica Thunb.	*Osmundaceae*	*Osmunda*			14.31	
Tetracera sarmentosa (L.) Vahl	*Dilleniaceae*	*Tetracera*				5.33

Note: Only plant species with *IV* greater than 5% were counted among different stands.
